# Impact of Agricultural Mechanization Level on Farmers’ Health Status in Western China: Analysis Based on CHARLS Data

**DOI:** 10.3390/ijerph20054654

**Published:** 2023-03-06

**Authors:** Huaquan Zhang, Zhenyao Yang, Yidan Wang, Martinson Ankrah Twumasi, Abbas Ali Chandio

**Affiliations:** College of Economics, Sichuan Agricultural University, Chengdu 611130, China

**Keywords:** agricultural mechanization, health status, agricultural machinery extension

## Abstract

Agricultural mechanization is an important component of agricultural modernization, as it contributes to the improvement of agricultural technology and the rapid transformation of agricultural development. However, research on the connection between agricultural mechanization and farmers’ health status is scarce. Thus, using the 2018 China Health and Retirement Longitudinal Survey (CHARLS) data, this study explored how agricultural mechanization can affect farmers’ health. OLS and 2SLS models were used for the study’s analysis. Furthermore, we used a PSM model to check the robustness of our analysis. The findings showed that: (1) the current state of agricultural mechanization in western China harms the health of rural residents; (2) agricultural mechanization can mitigate the adverse effects on health by increasing farmers’ living expenditure and improving their living environment; and (3) agricultural mechanization’s effects on farmers’ health are regionally and income-heterogeneous. Agricultural mechanization has a more significant impact on health in Tibetan areas and high-income regions. It has an almost minimal effect in non-Tibetan and low-income areas. This paper suggests approaches that can be used to encourage the rational development of agricultural mechanization and improve rural populations’ health.

## 1. Introduction

Health has long been considered critical in establishing national and international societies. At the international level, world leaders agreed on a political declaration on universal health coverage in September 2019 at the United Nations General Assembly. In China, since the publication of the Health China 2030 plan, health issues have become the center of national attention, and the report of the 19th National Congress of the Communist Party of China (CPC) proposed “implementing the health China strategy”. The western area is critical to the development of a healthy China, but its health lags well behind the eastern and central regions [1]. Due to the lack of health infrastructure and qualified human resources in the western rural areas, the development level of rural public health services in the western part of China shows a distribution pattern of “high in the middle”, “middle in the east”, and “low in the west” in terms of service efficiency [2]. As a result, the Chinese government and academics have prioritized the western region’s health issues.

According to previous research, health status is influenced by various factors, including environmental, educational, and economic factors. Katsikas’s study concluded that social circumstances, behavioral patterns, environmental exposure, and genetic predisposition significantly impact health [3]. Some studies consider that environmental sustainability, such as biodiversity and greenhouse gas emissions, may affect human health through food security [4,5]. The widespread use of pesticides has increased pesticide residues in food and drinking water, and pesticide use activities have caused several accidental poisonings [6]. Even routine pesticide use can pose significant health risks to farmers in the short and long term and degrade the environment [6,7,8,9]. Furthermore, economically, people with inadequate financial resources are less likely to have access to health facilities that allow them to improve their wellbeing [10,11]. According to [11], financial resources positively affect healthcare service utilization in China. Furthermore, educated people are more likely to understand the opportunity cost of not utilizing healthcare services [12]; thus, they are less likely to be affected by diseases and sicknesses that can be prevented.

Agriculture is the foundation of rural development in rural areas, and agricultural mechanization is critical to agricultural growth. Improving agricultural mechanization may be a critical aspect of the long-term development of agriculture because China is a huge agricultural country. Increasing investment in agricultural mechanization research can help to improve agricultural production and achieve sustainable development, increasing land productivity and food security [13]. The core of agricultural mechanization is the process of applying advanced agricultural machinery and equipment to gradually replace older, more primitive production tools, such as human and animal power; thus, obtaining agricultural machinery is an important issue in agricultural development. At the same time, agricultural mechanization is an important symbol of agricultural modernization and its basis [14]. Mechanization may have direct and indirect effects on farmers’ heath status. For example, some farming activities that are stressful or require manpower—and thus are detrimental to farmers’ health—can be undertaken with the use of farm machinery [15], implying that mechanization has a direct effect on health. Indirectly, using machinery for farming can improve farmers’ efficiency rates since it makes work simpler and faster while resulting in higher productivity [13]. As farmers’ productivity increases, they may obtain higher income, allowing them to access healthcare services that ensure healthy living. Agricultural modernization is an important part of China’s modernization, and the process of achieving socialist modernization with Chinese characteristics is closely related to the process of agricultural modernization [16]. While many factors can influence the health status of rural households, few studies have addressed this population’s health from the standpoint of agricultural mechanization, especially in rural areas of China, such as the western parts. However, as shown by the above scenario, a potential relationship exists between household health status and agricultural mechanization.

Since the agricultural industry in the western region is transitioning from a conventional, crude, high-consumption, and high-pollution industry to a high-yield, high-quality, and low-energy-use industry [17], we discuss and analyze the health status of rural residents in ten provinces in China’s western region, excluding Ningxia and Tibet, from the perspective of agricultural mechanization using data from the 2018 China Health and Retirement Longitudinal Survey (CHARLS), aiming to provide a foundation for improving health policies for the elderly. The study’s contributions are as follows. First, existing studies have discussed the health relationship somewhat adequately; however, few have explored the health–agricultural mechanization relationship. We here examine the mechanisms through which agricultural mechanization has an impact on health. Second, this paper uses suitable econometric models that address the endogeneity problem relating to the topic. Third, most of the existing literature focuses on China’s central and eastern regions, and there is not enough attention given to the underdeveloped western regions. In contrast, this study used a sample consisting of the less affluent western regions of China and aimed to present the current situation in the western regions and give corresponding suggestions. Fourth, in the discussion of regional heterogeneity, we chose to compare Tibetan regions with non-Tibetan regions. While most of the existing literature on Tibetan regions discusses the macro-developmental aspects of the overall area, we examined micro-individual aspects with the aim of revealing the differences between Tibetan and non-Tibetan regions in terms of individual health status.

The remainder of the paper is structured as follows. Section 1.1 provides a summary of relevant studies. The theoretical foundation and research hypotheses are described in Section 1.2. In Section 2, we provide the data sources, variable definitions, and model settings, and in Section 3, we analyze the empirical results. Section 4 summarizes the findings and examines the policy implications.

### 1.1. Literature Review

Many researchers have investigated the elements influencing population health, such as family factors, environmental factors, and individual characteristics. Family income, family size, and the education levels of family members are the critical family factors that determine health status. Income is generally a key driver of health in many nations worldwide. Martin discovered that absolute income affects health after investigating the association between self-rated health and income in persons aged 40–79 years in 21 countries [18]. In China, higher income has a considerable positive effect on health, and an increase in per capita household income can dramatically enhance older people’s health. Members of large families are healthier in western China, family size distribution is poor, and living together for several generations is conducive to the physical and emotional care of individual family members, particularly the elderly, which promotes health [19]. Grossman [20] developed a model of health needs and stated that increasing education would enhance the marginal productivity of medical care and time, hence improving health status. Zhao Zhong studied the health of rural Chinese people and discovered that a better education has a considerably favorable effect on health [21]. In terms of the environmental factors influencing health, the deterioration of the living environment is a significant variable affecting health in the western half of China [22]. In terms of living conditions, both drinking water quality and sanitation have significant impacts on rural residents’ health, and centralized water supply projects in western rural areas may be more vulnerable to insecurity [19]. Individual characteristics affecting health status include ethnicity, gender, and marital status. In the western region, where ethnic minorities are extensively scattered, ethnicity variables have a negative effect on health. Most relevant studies on gender and health have focused on empirical analyses of Asian countries. For example, Joung compared the depression status of men and women over 65 years old in South Korea and concluded that men, as the main source of household income, had less income and less financial security after their withdrawal from the labor market and that the relationship between wealth status and depression was significant among men, whereas women did not show this significant relationship. Gender inequalities in health exhibit themselves in differing lifespans, differences in dietary intake, and differences in mortality [23]. Women’s health is significantly worse than men’s in the country’s central and western regions [19]. Furthermore, unequal policy norms, the lower status of women in families, and women’s higher rates of participation in the unpaid or informal sector may make it difficult for women in less developed areas to access effective health services and may increase their health vulnerability [19]. Marital status might also have an impact on health [24]. The risk of death among single men and women is relatively higher than among married people because single people are more likely to be socially isolated, have lower social class and bad habits, etc., than married people [24].

Agricultural machinery occupies an important position in agricultural mechanization, and the evaluation of the development level of agricultural mechanization is essentially the scientific and rational evaluation and analysis of the economic and social effects produced by the use of agricultural machinery in the process of agricultural production [25]. In the development of agricultural mechanization, industrial structure and operation scale status are important environmental conditions, and economic and social benefits are the core of evaluation; i.e., with agricultural machinery operation as the basis, capacity as the guarantee, and benefits as the core, agricultural mechanization can be divided into three parts: the agricultural mechanization operation level, the comprehensive guarantee capacity of agricultural mechanization, and the comprehensive benefits of agricultural mechanization. The agricultural mechanization operation level is the basis of the evaluation of the development level of mechanization and the most critical index when evaluating the development level of agricultural mechanization, which involves continuous improvements in the mechanized operation levels for tillage, seeding, harvesting, plant protection, drainage, and irrigation [26]. Therefore, the possession and use of agricultural machinery represent the premise and the most important indicator of the development of agricultural mechanization, and they were also the main indicators for the evaluation of agricultural mechanization in this study. In recent years, as the government has aggressively promoted agricultural machinery purchase subsidies, the agricultural mechanization output of China’s various regions has grown reasonably quickly. However, in the western region of China, due to a variety of factors and the current overall agricultural production mechanization level, there is a certain gap and low efficiency in the use of agricultural machinery. The following are the primary reasons:

(1) Complex geographical environment and lack of high-standard farmland appropriate for large-scale mechanized production. For a long time, machine-farming roads in remote areas of the west have been too narrow and in disrepair due to land strip divisions, and in some locations, there are no machine-farming roads at all;
(2) Due to the small scale of rural collective economic organizations, certain village cadres are susceptible to financial monitoring, investment hazards, ideological capacity constraints, fear of accountability, and other factors preventing exemplary leadership from being fully realized;
(3) Farmers in the western region carry out production with very few skills and professional maintenance abilities. The aging of rural people engaged in agricultural production and the migration of the majority of the young labor force to cities have resulted in a shortage of people who can use agricultural machinery. Furthermore, the remaining middle-aged and elderly people have weak foundations for using and learning agricultural machinery skills;
(4) One of the major reasons for farmers’ substantial loss of efficiency when utilizing agricultural machinery is wear and tear of agricultural machinery [27,28,29,30,31].

### 1.2. Theoretical Framework and Hypotheses

In recent years, China’s agricultural machinery subsidies have continued to successfully boost farmers’ enthusiasm for acquiring machinery, and agricultural machinery usage has increased [32]. Farmers in the western area of China have less time to employ agricultural machinery in the natural farming process due to the existing scenario of low agricultural mechanization and the low efficiency of the agricultural machinery used. At the same time, farmers face enormous costs when purchasing and maintaining agricultural equipment. Moreover, agricultural equipment in the western region’s countryside can only be produced on a modest scale. According to this framework, the numerous expenses connected with mechanized equipment will only rise due to spontaneous market regulation, establishing a vicious circle [33]. Due to the fact that the income of rural inhabitants in the western region is lower than in other regions and because agricultural machinery is expensive, it is natural to cut out other living expenditures, which has a negative impact on health. Second, agricultural production personnel in the rural parts of western regions are aging and require a more significant foundation to help them learn the skills necessary to use agricultural machinery. Agricultural mechanization-related industries and talents cannot be developed, resulting in high equipment maintenance costs and, hence, reducing the attractiveness of agricultural mechanized equipment [34]. As a result, the current situation of agricultural machinery in the western region may impair farming efficiency and cause farmers to spend more time and energy on farming, negatively impacting their health.

**Hypothesis 1 (H1).**  *The current state of agricultural mechanization in the western portion of the country harms the rural population’s health*.

**Figure 1:**
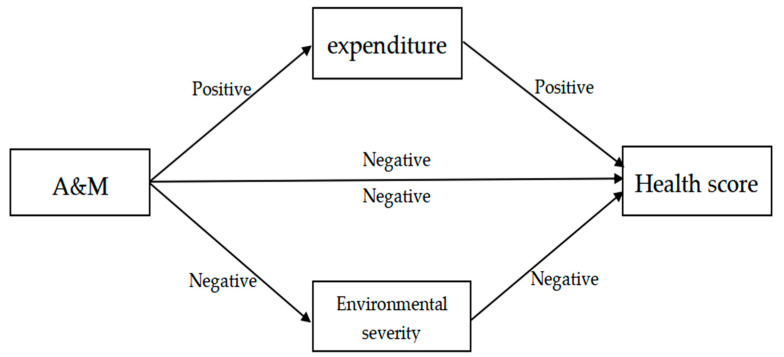
Mediating effects of living expenses and environmental severity.

Wang Yaqin et al. [35] discovered that agricultural mechanization considerably impacts farmers’ income. Greater income for farmers increases their consumption of material and spiritual goods, which improves their satisfaction in life and thus has a good impact on their health. At the same time, spending more on medical treatment will help farmers’ health improve. In addition, agricultural mechanization has improved the living environment of rural farmers, which is vital for achieving “village cleanliness”. Mechanized straw return and silage technologies, for example, have reduced sources of straw pollution, eliminated the problem of burning heaps, and cleansed the rural environment [36]. It has also been suggested that agricultural mechanization can reduce agricultural carbon emissions. He et al. argued that mechanized deep plowing and cutting promote low carbon efficiency [37]. Furthermore, as the living environment is essential to enhancing inhabitants’ health, agricultural mechanization can improve farmers’ health by optimizing the rural living environment. Figure 1 depicts the mediating effects of living expenses and environmental severity.

**Hypothesis 2a (H2a).**  *Increasing agricultural mechanization can boost farmers’ income and, as a result, their living expenditure while promoting improved health*.

**Hypothesis 2b (H2b).**  *Increasing agricultural mechanization can help minimize environmental harshness and promote better health*.

Due to the extensive territory of western China, where lifestyles, development status, and related regulations differ from region to region, the influences of agricultural modernization on the health status of rural populations may be heterogeneous. First, there are significant differences in economic conditions, lifestyles, and ideologies between Tibetan and non-Tibetan areas in the western provinces, and Tibetan areas are characterized by constraints on resources, infrastructure, the ecological environment, and the market economy in terms of agricultural development [38]. As a result, we anticipated that agricultural modernization’s influence on rural populations’ health would differ between Tibetan and non-Tibetan locations. Second, assessing the association between farmers’ usage of agricultural technology and health status revealed diverse effects on farmers’ income levels. Agricultural mechanization can greatly increase farmers’ income [39], so farmers who utilize agricultural machinery earn more than those who do not. Income levels also impact inhabitants’ health, and this disparity is particularly obvious in the high-income group.

**Hypothesis 3a (H3a).**  *The effects of agricultural mechanization on the health status of farm households will differ between Tibetan and non-Tibetan areas*.

**Hypothesis 3b (H3b).**  *Agricultural mechanization has a greater impact on the health of farmers with higher incomes*.

## 2. Materials and Methods

### 2.1. Data

The data in this research were obtained from the China Health and Retirement Longitudinal Survey (CHARLS), a large national household survey led by Peking University’s National Development Research Institute that focused on middle-aged persons aged 45 and up and their households. A baseline survey was conducted in 2011–2012 spanning 150 county-level units and 450 village-level units in 28 provinces and municipalities, with around 19,000 persons in 12,400 households. From 2013 through 2018, follow-up surveys were undertaken. The surveys requested specific information about individuals and households, and the results can be used in transdisciplinary research, such as economics and sociology.

Using the 2018 data from the China Health and Retirement Longitudinal Survey (CHARLS), 3834 rural households in the ten western provinces—Yunnan, Qinghai, Sichuan, Xinjiang, Inner Mongolia, Chongqing, Gansu, Guangxi, Shaanxi, and Guizhou—were chosen for the sample in this study.

### 2.2. Method

#### 2.2.1. Dependent Variables

**Figure 2:**
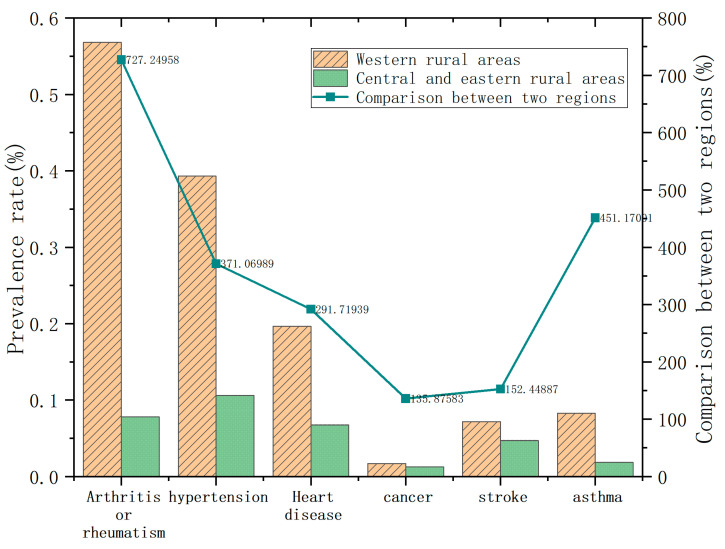
Prevalence of six diseases in China’s rural areas and comparison of prevalence in the western region and the central-eastern region in 2018.

The health score was the study’s outcome variable. To avoid the possibility of biased results from using subjective self-rated health status as the health indicator, we defined the health score variable as “Have you been told by a doctor that you have a certain disease?” in order to reflect the objective health status of rural middle-aged and elderly people. Acute diseases were included in the health score variable because they affect agricultural labor participation and time spent farming and because farmers are more likely to continue farming when they have chronic diseases [40]. Coile defines acute and chronic health shocks as comprising heart disease, cancer, stroke, and “three high” diseases, such as rheumatoid arthritis and renal disease [41]. In this study, after treatment was carried out, respondents were given a value of 1 if they were sick and 2 if they were not. The coefficient of variation approach was employed to provide weights for each ailment, with higher health scores signifying respondents’ better health state. Figure 2 depicts the likelihood of contracting the six diseases among 3834 farmers in western rural areas and 7694 farmers in central and eastern rural areas based on CHARLS 2018 data. Figure 2 shows that the numbers of people suffering from arthritis or rheumatism, hypertension, and heart disease are higher in rural areas across the country. The likelihood of contracting each disease is higher among farmers in western rural areas. The difference in prevalence rates between the two regions is greater than 100%, underlining the need to study farmers’ health in western rural areas.

#### 2.2.2. Key Variables

The total value of agricultural machinery was the main explanatory variable. Accelerating the integration of, innovation in, and application of advanced and practical technologies is a critical way to address the problems of the “three rural areas”. Accelerating R&D and upgrading agricultural machinery and equipment, as well as promoting agricultural mechanization, is a critical way to build a new type of agriculture. Agricultural mechanization can accelerate the progress of agricultural modernization in China and, to some extent, symbolizes the degree of agricultural modernization [42,43]. In this study, we employed the question, “Do you hold the following agricultural fixed assets, and how much are they worth now?” to compute the total value of the agricultural machinery held by survey respondents, thus quantifying their use of agricultural mechanization and level of agricultural mechanization.

#### 2.2.3. Mediator Variables

##### Living Expenses

The following question was used in this study: “How much does your household spend each month on average? All included are rent, food, clothing, communication expenditures, utilities, petrol, services, entertainment, daily essentials, and medical expenses”. The survey respondents’ total living expenses were computed.

##### Environmental Severity

The following questions were used in this study: “Does the house have running water?”, “Does the house have bathing facilities?”, “Does the house have piped gas or natural gas?”, and “Does the house have heating equipment?”. These four questions were used to assess rural populations’ living conditions. A value of 1 was assigned if a specific facility was available. If not, a value of 2 was assigned, and the values for the four facilities were put together to evaluate the respondents’ household living environment.

#### 2.2.4. Control Variables

This study used two variables of interest at the respondent and household levels as control variables. Gender, religious affiliation, marital status, education level, and hours worked in agriculture were used as control variables for responder characteristics. As control variables for household characteristics, the household per capita income, amount of agricultural subsidies received by the household, percentage of irrigable arable land area, and educational level of family members (respondents’ children) were used. The model variables and summary statistics are shown in Table 1.

Moreover, we chose the agricultural machinery use rate (IV) as an instrumental variable. The instrumental variable in this work was relatively exogenous because the rate of agricultural machinery utilization directly influences agricultural machinery promotion and the level of agricultural mechanization. However, the tools chosen have no direct impact on farmer health. Table 1 shows the model’s variables and summary statistics.

#### 2.2.5. Introduction to the Model

(1) Coefficient of variation method

The coefficient of variation approach was used in this paper to obtain the weights of each disease for the explanatory variable ${\mathrm{Health}}_{i}$. The coefficient of variation approach uses the information associated with each indicator to calculate the weight of the indicator, which is an objective weighting method that can reduce the influence of human factors and increase the accuracy of the results.

(1) $$V_{i}=\frac{\delta_{i}}{\bar{x_{i}}}$$

(2) $$W_{i}=\frac{V_{i}}{\sum_{i=1}^{n}V_{i}}$$

where ith is the coefficient of variation of the evaluation indicator; δ_i_ denotes the standard deviation; $\bar{x_{i}}$ is the mean value; and W_i_ is the weight of the ith evaluation indicator. In this study, we assigned weights to each disease based on the magnitude of the variation in the survey values for the 3834 rural residents for each disease. If an indicator’s coefficient of variation was more significant, this indicator had more explanatory power in determining health status. This indicator was then given a higher weight, and the specific weightings are shown in Table 2.

(2) Baseline return

The core of the model in this paper was the estimation of the effects of changes in the total value of agricultural machinery on population health as a reflection of the health-promoting effects of agricultural modernization. The explanatory variables were the composite measures of the six diseases mentioned above, which made it possible to measure individuals’ health status.

(3) $${\mathrm{Health}}_{i}=\alpha_{0}+\alpha_{1}{\mathrm{Agri}}_{i}+{\gamma X}_{i}+\varepsilon_{i}$$

The core explanatory variable in the above model is Agri_i_, which denotes the total value of the agricultural machinery owned by individuals. X_i_ represents the control variables and a set of other micro factors associated with resident i, such as irrigable rate, education level, highest education level of children, per capita household income, amount of agricultural subsidy, hours of agricultural work, gender, marriage, religion, and grazing. ε_i_ represents a set of randomly disturbed phrases.

(3) Analyzing possible mechanisms

This research also employed a mediating effects test to analyze the impact mechanism through estimation with the following model in order to investigate the influence channel linking the overall value of agricultural machinery and health.

(4) $$M_{i}=\beta_{0}+\beta_{1}{\mathrm{Agri}}_{i}+{\gamma X}_{i}+\varepsilon_{i}$$

(5) $${\mathrm{Health}}_{i}=\gamma_{0}+\gamma_{1}{\mathrm{Agri}}_{i}+\gamma_{2}M_{i}+{\gamma X}_{i}+\varepsilon_{i}$$

In this model, M_i_ represents a mediating variable, such as living expenses or environmental severity; α_1_ represents the total effect of the total value of agricultural machinery on health; γ_1_ measures the direct effect of the total value of agricultural machinery on health; β_1_ and γ_2_ represent indirect effects and the magnitude of indirect effects. If the coefficients α_1_, β_1_, and γ_2_ are significant, the mediating effect can be assumed to exist and be significant. Assume the bootstrap approach is used to test the significance of the mediating effect. In that situation, the mediating effect would be regarded to exist only if the bootstrap 95% confidence interval did not contain 0, indicating that the mediating impact was substantial.

## 3. Results and Discussion

### 3.1. Empirical Results Regarding the Impact of Agricultural Mechanization on Farmers’ Health

Table 3 displays the estimation results from the OLS and 2SLS. There are no control variables in column (1), and only the primary explanatory variables were included in the regression. According to the regression results, the total value of agricultural machinery has a substantial and positive effect on farmers’ health ratings at the 10% level, demonstrating that agricultural mechanization promotes population health. Given that the dependent variable in this research assessed farmers’ health condition and its coefficient did not show the degree of promotion of farmers’ physical function, only the coefficient’s direction of change and significance level should be considered. In column (2), all control variables are combined, and the final findings show that the role of the total value of agricultural machinery in influencing health did not change significantly. Except for the number of agricultural subsidies and road conditions, all of the non-individual characteristic variables among the control variables had positive effects, while the individual’s education level had no effect on his or her health status, and the rest of the variables were very significant.

Given the endogeneity of agricultural machinery value, which could have introduced bias into the original OLS, 2SLS was evaluated by using the utilization rate of different value levels of agricultural machinery at the village level as an instrumental variable. We followed Chyi and Mao [44] in this study and utilized the weak instrumental variable test to examine the plausibility of the instrumental factors, the results of which are reported in Table 4. The Kleibergen–Paap rk Wald F statistic was greater than the critical value of the Stock–Yogo weak identification test at the 10% level in the weak identification of instrumental variables test. The foregoing experiments demonstrate that the instrumental factors employed were quite successful. The overall value of agricultural machinery was found to have a substantial negative influence on health status compared to OLS, and shown in column (3) of the second-stage regression findings, while the effect of the number of agricultural subsidies on health was no longer significant and production inputs changed from having an insignificant to a significant positive effect on health. This shows that the number of agricultural subsidies had no effect on farmers’ physical health but production inputs had a positive impact on health, and the estimates for the remaining variables were approximately equivalent to the OLS estimates. Columns (3) and (4) show the regression results comparing the impact of agricultural mechanization on health in the western region and nationwide, respectively. The regression results were roughly convergent, with agricultural subsidies and individual education level re-emerging at the national level. From the national perspective, agricultural mechanization had a significant negative effect on health, but the magnitude of its effect was much smaller than the negative effect in the western region, and the marginal effects of both differed more in terms of income level and livestock farming.

To summarize, the overall value of agricultural machinery had a major impact on farmers’ health in China’s rural districts. The impact was especially prominent in the western region. At the national level, the high purchase price of farm machinery, the small size of farmland, the fragmentation of land, and the lack of machinery training make most farmers less willing to use farm machinery even if they are aware of the benefits of using farm machinery, making it difficult for farm machinery to have the desired effect [45]. However, in the western region, the influences of geographical conditions, labor force structure, and resource and environmental issues should also be considered. At present, the use rate of agricultural machinery in hilly mountainous areas, the arable land of which accounts for one third of China’s arable land, is only about 50%, which is more than 30 percentage points lower than that in plain areas [46], while in the western region, especially in the southwest, the total area occupied by mountains and hills accounts for more than 90% of the local area, and the plain area is less than 5%; in this case, idle agricultural machinery and shortages in machinery are more serious. Secondly, the essence of agricultural mechanization is the substitution of labor. The urbanization process in China is currently resulting in labor transfer from rural to urban areas, and the more scarce the labor force is, the stronger the demand for mechanization will be [47]. However, the scarcity of arable land and the relative surplus of rural labor are notable problems in western regions, leading to the low willingness of farmers to use agricultural machinery, which, in turn, affects the efficiency of the use of agricultural machinery. Furthermore, China’s agricultural development faces prominent resource and environmental problems, especially in the western rural areas where the natural ecosystem is relatively fragile. In the west, the problems include the low carrying capacity of arable land and the very limited environmental carrying capacity, heavy reliance on pesticides, extensive pollution from untreated livestock and poultry excreta through the discharge of water [28], and livestock polluting the environment by causing air pollution, land pollution, and water pollution. In each watershed, the impact of livestock pollution on water quality has exceeded those of residential life, agriculture, township work, and catering [48]. In the western part of the country, livestock farming is more concentrated, which can lead to the deterioration of the health of farm households. The irrigable rate, per capita household income, duration of agricultural employment, and production input all positively impact health. The higher the per capita household income is, the more likely farm it is that households will invest in healthcare to improve their health. With more investment in agricultural production, production operations reach a larger scale; the more mature the corresponding production system is, the more efficient production becomes, and the more agricultural income increases, thus positively impacting health. Farmers’ education level was found to have no effect on their health, and the higher the education levels of farmers’ children were, the better their health was. Most middle-aged and older adults in western rural areas, who generally have low education levels, have a very limited ability to learn about using agricultural machinery without relying on others. They are likely to know little about agricultural machinery even when taught through government interventions. In the case of unregulated use of farm machinery—or even if farmers own farm machinery but leave it idle—the farm machinery will not be as effective as it should be and may even have negative effects, which may be offset by the subtle influence of children on the middle-aged and elderly. The higher the education levels of children are, the more likely they are to be aware of the benefits of using farm machinery for the middle-aged and elderly and to take the initiative to acquire knowledge related to using and maintaining farm machinery. Future interventions should further encourage middle-aged and older adults to use agricultural machinery in their daily lives, popularize agricultural machinery and instruct middle-aged and elderly farmers in its use, improve middle-aged and elderly farmers’ knowledge of agricultural machinery, and help middle-aged and elderly farmers develop a positive awareness regarding the use of agricultural machinery. Furthermore, such interventions should correct middle-aged and elderly farmers’ methods of using agricultural machinery, and instruct them in how to undertake timely maintenance and repair of agricultural machinery, which should significantly reduce the possibility of farmers’ unconscious loss of agricultural machinery and improve the efficiency of farmers’ use of agricultural machinery. The insignificant effect of agricultural subsidies on farmers’ health is due to the lower productivity level in western China and the backward agricultural infrastructure and land conditions compared to the central region. The results are in line with a study that revealed that the marginal contribution of agricultural subsidies to farmers’ income in the western region was lower compared to other regions of China [49]. Farmers who had partners were healthier than those who did not have partners, farmers who had religious beliefs were healthier than those who did not have such views, women were less healthy than men, and farmers who raised animals were less healthy than those who raised crops. Farmers engaged in animal husbandry typically had a larger grazing area than those engaged in agricultural production, implying that the scope of their labor activities was much broader. A higher value for road condition represents worse road conditions, and it was significantly negatively correlated with health status because, when road conditions are better, traffic will be more convenient, and it will be more convenient for the rural middle-aged and elderly to go out for medical treatment when they are sick. Therefore, better road conditions have a positive effect on the health status of rural elderly.

### 3.2. Robustness Tests

In addition to using instrumental variables, propensity score matching (PSM) can also be used to alleviate endogeneity difficulties. In contrast to instrumental factors, propensity score matching primarily addresses endogeneity issues induced by the sample selection. To estimate propensity scores, the PSM model often uses a logit model. The dummy variable “whether to utilize farm machinery” was used in this study. The sample classified as “using farm machinery” served as the experimental group, while the sample classified as “not using farm machinery” served as the control group. Finally, the effect of agricultural mechanization on health was assessed by calculating the difference in agricultural equipment use between the control and experimental groups; i.e., the average treatment effect (ATT). In this study, three matching approaches were used: (1) near-neighbor matching—1:4 matching (k = 4) was utilized; (2) caliper matching—Due to the large sample gap, near-neighbor matching could have led to bias, so caliper matching was included in this work, and the caliper range was set as 0.01; (3) kernel matching—in the kernel matching process, a secondary kernel was used.

**Figure 3:**
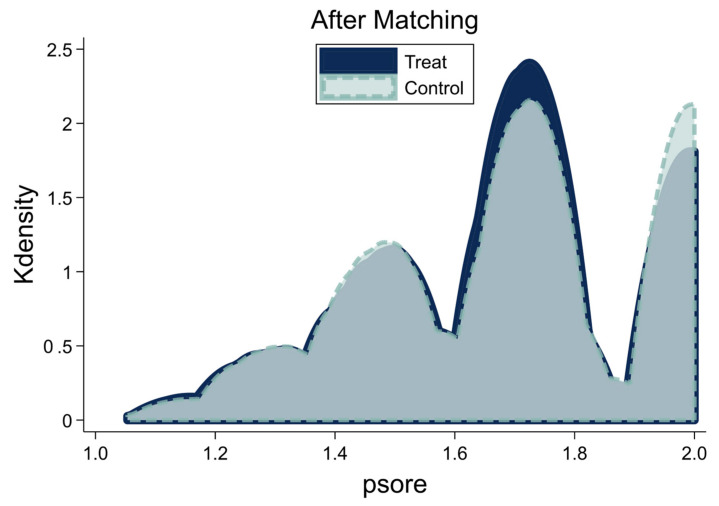
Density function after propensity score matching.

The nearest-neighbor matching approach was used to examine the matching effect with the PSM method by displaying the probability density function of the control and experimental groups: the greater the overlap between the control group and the experimental group, the better the matching quality. The overlap interval in the tendencies of the scores for the experimental group using farm machinery and the control group not using farm machinery was more extensive, as illustrated in Figure 3. The probability density function followed the same pattern, demonstrating that the control variables of the two groups were closer in several respects after matching, and the matching effect was stronger. The remaining two matching methods’ probability density function graphs were similar to the above situation and will not be shown here. To meet the matching criteria, the standardized deviation after matching had to be less than 10%, and all *p*-values had to be greater than 0.05. The standardized deviations after matching were less than 5% for all three approaches, all *p*-values were greater than 0.05, and the smoothness test confirmed that the matching methods were appropriate. Table 5 displays the specific outcomes.

The three methods used for the propensity score matching model eventually produced ATT effects that were significantly negative at the 10% level, confirming that agricultural mechanization has a significant negative effect on the health of farmers in the western region and demonstrating the robustness of the analytical findings.

### 3.3. Analysis of the Mechanism of Intermediary Action

#### 3.3.1. Mediating Effects of Living Expenses

Table 6 shows the findings from the test regarding the mediating influence of living expenses. We began by conducting a preliminary test of the mediating effects in three steps. Column (1) depicts the inhibitory effect of agricultural machinery value on health. Column (2) shows that the coefficient between the total value of agricultural machinery and subsistence expenditure was significantly positive, and it can be assumed that the higher the total value of agricultural machinery is, the more income and, thus, subsistence expenditure can be increased as a result of increases in crop productivity. The regression coefficient for the total value of agricultural machinery shown in column (3) was still significantly negative at the 5% level. In comparison, at 5%, the coefficient for living expenses was notably positive. The indirect effect was the inverse of the direct effect, demonstrating the “masking effect” and indicating that the entire value of agricultural machinery can affect health by influencing living expenses. The theory of the “masking effect” would argue that an increase in the total value of agricultural technology could lead to an increase in subsistence expenditure, which, to some extent, would prevent farmers’ health from deteriorating further due to insufficient subsistence expenditure. The findings of the bootstrap test are also provided in Table 7 to further corroborate the mediating function of subsistence expenditure. The results show that the indirect effect was 0.00213621 and the direct effect was −0.08432967. The intervals in the table are the bias-corrected confidence intervals of [0.0001072, 0.0064038] and [−0.1492562, −0.0135406], respectively, with 95% confidence intervals that do not contain zero. The “masking effect” was demonstrated and consistent with hypothesis H2 of this research.

#### 3.3.2. Mediating Effects of Environmental Severity

Table 8 shows the test findings for the degree of environmental harshness, and the verification processes were similar to those mentioned above for the mediation impact of living expenses. Column (2) shows that the coefficient between the total value of agricultural machinery and the degree of environmental harshness was significantly negative, implying that the greater the total value of the agricultural machinery held by farmers, the more probable it is that their living environment will be significantly improved. In column (3), the regression coefficients for the total value of agricultural machinery and the degree of the poor environment were all significantly negative at the 1% level. The indirect impact showed a “masking effect” that was the inverse of the direct effect. The increase in the total value of agricultural machinery improved farmers’ living environment, to some extent preventing the continuous deterioration of farmers’ health status due to the poor living environment and thus offsetting part of the negative effect of agricultural mechanization on health. Table 9 summarizes the findings from the bootstrap test. The indirect effect was 0.00484275, while the direct effect was −0.08703621, both of which did not have a 0 in their 95% confidence intervals. In accordance with hypothesis H2b of this work, the indirect effect had the opposite sign to the direct effect, and the masking effect was validated for environmental harshness.

### 3.4. Heterogeneity Effect

#### 3.4.1. Regional Heterogeneity

Exploring the heterogeneity between regions was considered critical for the investigation of the subject of this study, given the disparities in the health statuses of rural populations in different geographical locations of western China. The CHRALS 2018 database only contains data from three localities among the Tibetan areas in the four western provinces—namely, Liangshan Yi Autonomous Prefecture, Chuxiong Yi Autonomous Prefecture, and Ganzi Tibetan Autonomous Prefecture—and does not include all Tibetan areas. Furthermore, the population share of the Tibetan areas is small, so the sample size gap between Tibetan and non-Tibetan areas is large. For group regression in this paper, the sample was separated into Tibetan and non-Tibetan regions, and the results are reported in Table 10. In addition to the key explanatory variable—the total value of agricultural machinery—the irrigable rate [50], which also plays an important role in agricultural development, was included as a control variable in the regression. According to the regression results, the influence of agricultural mechanization on the health status of rural populations was more significant in Tibetan areas. In fact, in the western Tibetan region, there are many areas that are part of national nature reserves. Due to the high level of environmental protection in the region, the harsh natural environment, and the distribution of mountains and valleys located in remote areas, the process of agricultural machinery promotion is more difficult and affects less agricultural machinery. This is in addition to the ordinary agricultural machinery promotion difficulties, and Tibetan areas are mostly high-altitude areas, with high terrain, plateaus, and mountains. Furthermore, agricultural infrastructure construction is lagging, there are no big cities to rely on for support in development, the region lacks roads that would allow the normal operation of agricultural machinery, and there is a complex cultural environment, subject to the effects of language, culture, religion, and other objective factors, as well as many other constraints [51,52]. As a result, compared to non-Tibetan areas, the use of agricultural machinery in Tibetan areas may be less efficient while having a greater detrimental impact on farmer health.

#### 3.4.2. Income Heterogeneity

Given the disparities in rural inhabitants’ health statuses caused by farm household income, it was judged to be vital to analyze the heterogeneity brought about by high and low income. As a result, the median per capita household income was used as the judgment criterion in this study. Income was classified in the high-income category if it was greater than the median. Otherwise, it was classed as low-income. The two categories were individually regressed, and the regression results are displayed in Table 11. According to the regression results, the overall value of agricultural machinery was significant only at the high-income level. In contrast, while significant in both groups, the irrigable rate had a stronger boosting effect in the high-income group. In comparison to the low-income level, the significance threshold was raised from 5% to 1%. This indicated that the effect of agricultural mechanization on rural residents’ health was more pronounced in the high-income-level group, which was because, in the context of the negative effect of agricultural mechanization level on rural residents’ health in the western region, the high-income-level group was found to be more likely to purchase agricultural machinery, resulting in the heterogeneous effect being significant in the high-income-level group.

## 4. Conclusions and Implications

### 4.1. Conclusions

Based on the theoretical study and empirical assessment, the following conclusions were reached.

First, according to the regression analysis, agricultural mechanization in the western area of China reduced the health levels of rural residents in the current state of agricultural equipment development. Furthermore, the control variables agricultural subsidy amount, gender, and grazing significantly and negatively impacted farmers’ health. In contrast, control variables such as the irrigable rate, highest level of education of children, and per capita household income had significant and favorable impacts on farmers’ health.

Second, the degree of agricultural mechanization was found to affect the health of rural inhabitants through farmers’ living expenses and living environment in accordance with the mediating effect. Both high living costs and a terrible environment had “masking effects”, masking the negative impacts of agricultural mechanization on farmers’ health in the western region.

Third, heterogeneity analysis revealed that agricultural mechanization’s effect on rural populations’ health was stronger in Tibetan areas and among higher income groups. This indicated that the more agricultural machinery high-income Tibetan and western-area residents own relative to non-Tibetan and low-income western-region residents, the poorer their health is.

### 4.2. Implications

This study has specific practical significance and shows that China needs to promote the development of agricultural mechanization in specific ways depending on the state of agricultural development in various regions and create policies for the development of agricultural machinery based on local conditions. Otherwise, such interventions may be detrimental to the health of local farmers. Farmers’ bad health will reduce their agricultural work time and energy, making agricultural machinery more efficient and creating a vicious circle that will result in increased agricultural development. According to the findings of this study, the government and key authorities should implement policies that encourage the rationalization of agricultural machinery use and increase the efficiency of agricultural machinery in different regions of China. First, the western area should be encouraged to accelerate the execution of high-quality agriculture projects. The western region’s farmland foundation is poor, and much farmland is inaccessible to agricultural machines. Project performance evaluations of high-quality farmland should be comprehensively undertaken to achieve the intended implementation effects and ensure the long-term benefits of livelihood projects [53], as well as to fundamentally solve the dilemmas relating to agricultural mechanization production and agricultural machinery use in the western region. Second, agricultural machinery talents should be aggressively introduced in the western region to improve local farmers’ technical training. By hiring from various agricultural colleges and universities, the local government may be able to absorb high-quality experts from the start to work in agricultural machinery management. Furthermore, the government should set up special machinery maintenance centers and provide corresponding agricultural machinery maintenance subsidies to reduce the cost of agricultural machinery maintenance and arrange for corresponding technical training, such as classroom teaching, practical teaching, on-site teaching, and so on, regarding the safe use, maintenance, and repair of standard agricultural machinery.

From the standpoint of the intermediary effects, if agricultural machinery is rationally developed and used in the western region of China, it could improve farmers’ income, living expenses, living environment, and quality of life, thus improving their health. As a result, the proper marketing and usage of agricultural machinery would be extremely beneficial. From the standpoint of heterogeneity, the government should, first and foremost, pay more attention to the development of agricultural mechanization in Tibetan areas, formulate more policies suitable for the development of agricultural machinery in Tibetan areas, and reduce the adverse effects of agricultural machinery on farmers’ health in Tibetan areas. Second, as high-income households use agricultural machinery more than low-income households, greater emphasis should be placed on this group to strengthen their understanding of agricultural machinery use and their ability to fix faults while using agricultural machinery. For low-income individuals, the government should provide subsidies to motivate them to use agricultural mechanization, which would ensure quality health status among them, all other things remaining constant. Furthermore, although the government’s agricultural subsidies seem to have a positive effect across the whole country, in the western region, the subsidies cannot play a proper role, so the government must adjust and improve the subsidy mechanism for the western region. In addition, we saw that religion positively affected the outcome variable. This implies that the government should organize health talk programs through religious activities to disseminate health information to religious group members.

These recommended policies may help mitigate the negative impact of current agricultural mechanization use in China’s western region on the health of rural residents, allowing farmers in the western region to engage in agricultural activities in a healthier state and thereby improving China’s agricultural businesses.

### 4.3. Limitation of the Study

This study had some shortcomings that should be addressed in future investigations. First, we only analyzed the situation in the western region and compared it with the overall national situation, but we did not include a relevant discussion of other regions. Due to China’s enormous population, the sample size for this study may have been rather modest. Future studies could broaden the study’s geographical scope and sample size to validate this paper’s results further. Second, this study relied on cross-sectional data from 2018, but the effect of agricultural mechanization on health status may be dynamic. Future research might examine the dynamic relationship using panel data from successive surveys. Third, the degree of agricultural mechanization may have other implications for the health of rural populations in China’s eastern and western regions, as well as in other countries. Future research could look into the consequences of agricultural modernization for people’s health in different countries. Finally, future studies could analyze the effects of other factors, such as environmental and educational effects, on rural household health from a broader perspective. We were limited based on the data available.

## Figures and Tables

**Table 1:** Definitions and data descriptions for variables in the model.

Variable	Definition	Mean	SD
	Dependent Variable		
Health score	The higher the score, the healthier the body is	1.70	0.241
	Key variables		
A&M	Total present value of agricultural fixed assets owned by the respondent’s household, including tractors, threshers, harvesters, pumpers, processing machines, seeders, and agricultural aircraft (million)	0.12	0.908
	Mediate variables		
Expenditure	Amount spent by the respondent’s household in the past month, including rent, food, clothing, communication expenses, utilities, fuel, service expenses, entertainment expenses, daily necessities, medical expenses, etc. (million)	0.14	0.202
Environmental severity	Evaluation of the living environment of the respondent’s household; the lower the score, the better the living environment (including whether the living environment has a toilet, electricity, running water, heating facilities, bathing facilities, piped gas or natural gas, a telephone, broadband Internet access, and an air purifier)	15.45	1.683
	Control variables		
Irrigable rate	Percentage of the irrigable arable land area owned by the respondent’s household versus the area of arable land received in the respondent’s collective allocation	0.37	0.438
Education	Respondent’s education level (1 = no education, 2 = elementary to junior high school education, 3 = high school education and above)	1.47	0.590
Children’s education	Highest level of education that the respondent’s children have received (1 = no education or formal education, 2 = did not finish elementary school, 3 = private school, 4 = graduated from elementary school, 5 = graduated from middle school, 6 = graduated from high school, 7 = graduated from secondary school (including secondary teacher training and vocational high school), 8 = graduated from college, 9 = bachelor’s degree, 10 = master’s degree, 11 = doctoral degree)	5.44	1.995
Production input	Total amount of money invested by the respondent’s household in the past year for agricultural and forestry production, including seeds, fertilizers, farm fertilizers, pesticides, plastic films, hired labor, land rent, rent other than land rent, irrigation, fuel, transportation, processing, market expenses, etc. (million)	0.204	0.371
Working hours	Time respondents worked in agriculture in the past year (hours)	654.78	898.029
Income	The average income of the respondent’s household in the past year (including income from all work wages and retirement pensions)	2.30	2.727
Agricultural subsidy	Agricultural subsidies received from the government during the past year (million)	0.05	0.166
Herding	0 if working in animal husbandry, 1 otherwise	0.03	0.183
Gender	1 if the respondent is male, 2 if the respondent is female	1.53	0.499
Religion	1 if the respondent is religious, otherwise 2	1.90	0.294
Marriage	1 if the respondent is married, otherwise 2	0.84	0.367
Village road condition	Traffic road conditions in the respondent’s village (1 = paved road, 2 = pathway/dirt/unpaved road, 3 = sandstone road)	1.81	0.744
Utilization rate (IV)	Utilization rates for farm machinery of different value levels in the respondent’s village (value levels were categorized as unused farm machinery, farm machinery less than USD 1000, farm machinery between USD 1000 and USD 10,000, and farm machinery over USD 10,000)	0.62	0.275
Observations	3834		

**Table 2:** Health status (Health_i_) weight vectors for the six diseases.

	High Blood Pressure	Cancer	Heart	Stroke	Arthritis or Rheumatism	Asthma
Weights	25.02%	5.34%	18.04%	10.99%	28.81%	11.80%

**Table 3:** Regression results for the impact of agricultural mechanization on farmers’ health.

Variable	(1) OLS	(2) OLS	(3) 2SLS (Western Region)	(4) 2SLS (National)
A&M	0.007 *	0.008 *	−0.082 **	−0.035 **
	(1.67)	(1.79)	(−2.44)	(−2.29)
Irrigable rate		0.029 ***	0.025 ***	0.017 ***
		(3.28)	(2.83)	(3.53)
Education		0.005	0.005	0.021 ***
		(0.71)	(0.74)	(5.28)
Children’s education		0.005 ***	0.008 ***	0.003 **
		(2.69)	(3.56)	(2.41)
Production input		0.017	0.038 ***	0.025 ***
		(1.55)	(2.83)	(3.77)
Income		0.003 **	0.004 **	0.012 ***
		(2.15)	(2.45)	(9.27)
Agricultural subsidy		−0.066 ***	−0.013	−0.075 ***
		(−2.83)	(−0.44)	(−3.70)
Working hours		0.000 ***	0.000 **	0.000 ***
		(2.69)	(2.57)	(4.89)
Gender		0.052 ***	−0.049 ***	−0.027 ***
		(4.85)	(−6.40)	(−5.73)
Marriage		0.077 ***	0.057 ***	0.052 ***
		(5.74)	(5.21)	(8.06)
Religion		−0.048 ***	0.061 ***	0.030 ***
		(−6.31)	(4.18)	(4.14)
Herding		−0.092 ***	−0.083 ***	−0.019 ***
		(−4.20)	(−3.76)	(−3.93)
Village road condition		−0.018 ***	−0.018 ***	−0.019 ***
		(−3.39)	(−3.38)	(−6.71)
Constant	1.704 ***	1.560 ***	1.578 ***	1.632 ***
	(435.05)	(48.15)	(47.72)	(86.84)
R-squared	0.000	0.055	0.056	0.045
F	2.776	18.215	18.439	42.365
Observation	3834.000	3834.000	3834.000	11,364.000

Robust standard errors are in parentheses; * *p* < 0.10, ** *p* < 0.05, *** *p* < 0.01.

**Table 4:** Weak instrumental variables test.

Kleibergen–Paap rk LM statistic	20.848	(0.000)
Kleibergen–Paap rk Wald F statistic	21.313	{16.38}

**Table 5:** ATT estimation results for PSM.

Projects	Processing Group	Control Group	ATT	T-Statistic
Nearest-neighbor matching (k = 4)	1.7045125	1.72258025	−0.018067746	−1.92 *
Caliper match (radius = 0.01)	1.70365251	1.71947695	−0.015824441	−1.82 *
Nuclear matching (secondary nuclear)	1.70410823	1.719122	−0.015013772	−1.75 *

Robust standard errors are in parentheses; * *p* < 0.10.

**Table 6:** Mediating effects of living expenses.

Variable	Health Score	Expenditure	Health Score
A&M	−0.082 **	0.053 *	−0.084 **
	(−2.44)	(1.85)	(−2.50)
Expenditure			0.040 **
			(2.12)
Constant	1.578 ***	0.132 ***	1.573 ***
	(47.72)	(4.69)	(47.45)
R-squared	0.056	0.027	0.057
Control variables	Yes	Yes	Yes
Observation	3834.000	3834.000	3834.000

Robust standard errors are in parentheses; * *p* < 0.10, ** *p* < 0.05, *** *p* < 0.01.

**Table 7:** Living expenses bootstrap test.

Variable		Coefficient	Standard Error	Z	*p*	95% Confidence Interval	
Expenditure	Indirect effect	0.00213621	−0.0000577	1.45	0.147	0.0001072	0.0064038
	Direct effect	−0.08432967	0.0007005	−2.44	0.014	−0.1492562	−0.0135406

**Table 8:** Mediating effects of environmental severity.

Variable	Health Score	Environmental Severity	Health Score
A&M	−0.082 **	−0.470 **	−0.087 ***
	(−2.44)	(−1.98)	(−2.59)
Environmental severity			−0.010 ***
			(−4.49)
Constant	1.578 ***	15.736 ***	1.740 ***
	(47.72)	(67.68)	(35.57)
R-squared	0.056	0.046	0.061
Control variables	Yes	Yes	Yes
Observation	3834.000	3834.000	3834.000

Robust standard errors are in parentheses; ** *p* < 0.05, *** *p* < 0.01.

**Table 9:** Environmental severity bootstrap test.

Variable		Coefficient	Standard Error	Z	*p*	95% Confidence Interval	
Environmental severity	Indirect effect	0.00484275	0.00274018	1.77	0.077	0.0003028	0.01112
	Direct effect	−0.08703621	0.0011498	−2.61	0.009	−0.1512689	−0.0225708

**Table 10:** Results of the regional heterogeneity test.

Variable	Non-Tibetan	Tibetan Area
A&M	−0.041	−0.393 ***
	(−1.15)	(−3.88)
Irrigable rate	0.025 **	0.010
	(2.57)	(0.44)
Constant	1.640 ***	1.501 ***
	(39.85)	(20.47)
R-squared	0.037	0.207
Control variables	Yes	Yes
Observation	3275.000	559.000

Robust standard errors are in parentheses; ** *p* < 0.05, *** *p* < 0.01.

**Table 11:** Results of the income heterogeneity test.

Variable	Low-Income Groups	High-Income Groups
A&M	−0.045	−0.109 **
	(−0.92)	(−2.33)
Irrigable rate	0.025 *	0.027 **
	(1.95)	(2.16)
Constant	1.581 ***	1.561 ***
	(31.38)	(34.92)
R-squared	0.042	0.069
Control variables	Yes	Yes
Observation	1916.000	1918.000

Robust standard errors are in parentheses; * *p* < 0.10, ** *p* < 0.05, *** *p* < 0.01.

## Data Availability

The data were released to the researchers without providing access to any personal data. Data access link: http://charls.pku.edu.cn/ (accessed on 5 May 2022).
